# Advancements in oncology nursing: Embracing technology-driven innovations

**DOI:** 10.1016/j.apjon.2024.100399

**Published:** 2024-02-08

**Authors:** Guolong Zhang, Xuanhui Liu, Yingchun Zeng

**Affiliations:** Respiratory Intervention Center, The First Affiliated Hospital of Guangzhou Medical University, Guangzhou, China; Department of Industrial Design, Hangzhou City University, Hangzhou, China; School of Medicine, Hangzhou City University, Hangzhou, China

Cancer represents a significant threat to global human health and longevity. In 2020, approximately 19.3 million new cases of cancer and 10 million fatalities were recorded worldwide.^1^ Innovations and advancements in medical technology have introduced novel approaches to prolong the survival of patients with cancer. These advancements offer both opportunities and challenges in the field of oncology nursing.^2^ The incorporation of digital technologies such as telehealth and remote monitoring, mobile technology applications, electronic health records, simulation technologies such as virtual reality (VR), and other artificial intelligence (AI) technologies, has become a cutting-edge area in oncology nursing research. Recent studies published in this Journal indicate that these technological advancements positively impact cancer patient care in areas of health conduction follow-up, health education, symptom management, and interventions.^3^, ^4^, ^5^, ^6^

The challenges in nursing care for patients with cancer are multifaceted and continuously evolving.^7^ While technological innovation might enhance certain aspects of cancer care, it may be unable to address all aspects cancer nursing care. Hence, to systematically enhance the quality of cancer care, it is imperative for clinical practice to integrate these technologies, establishing a multidimensional cancer nursing care system as proposed in Fig. 1.

**Fig. 1:**
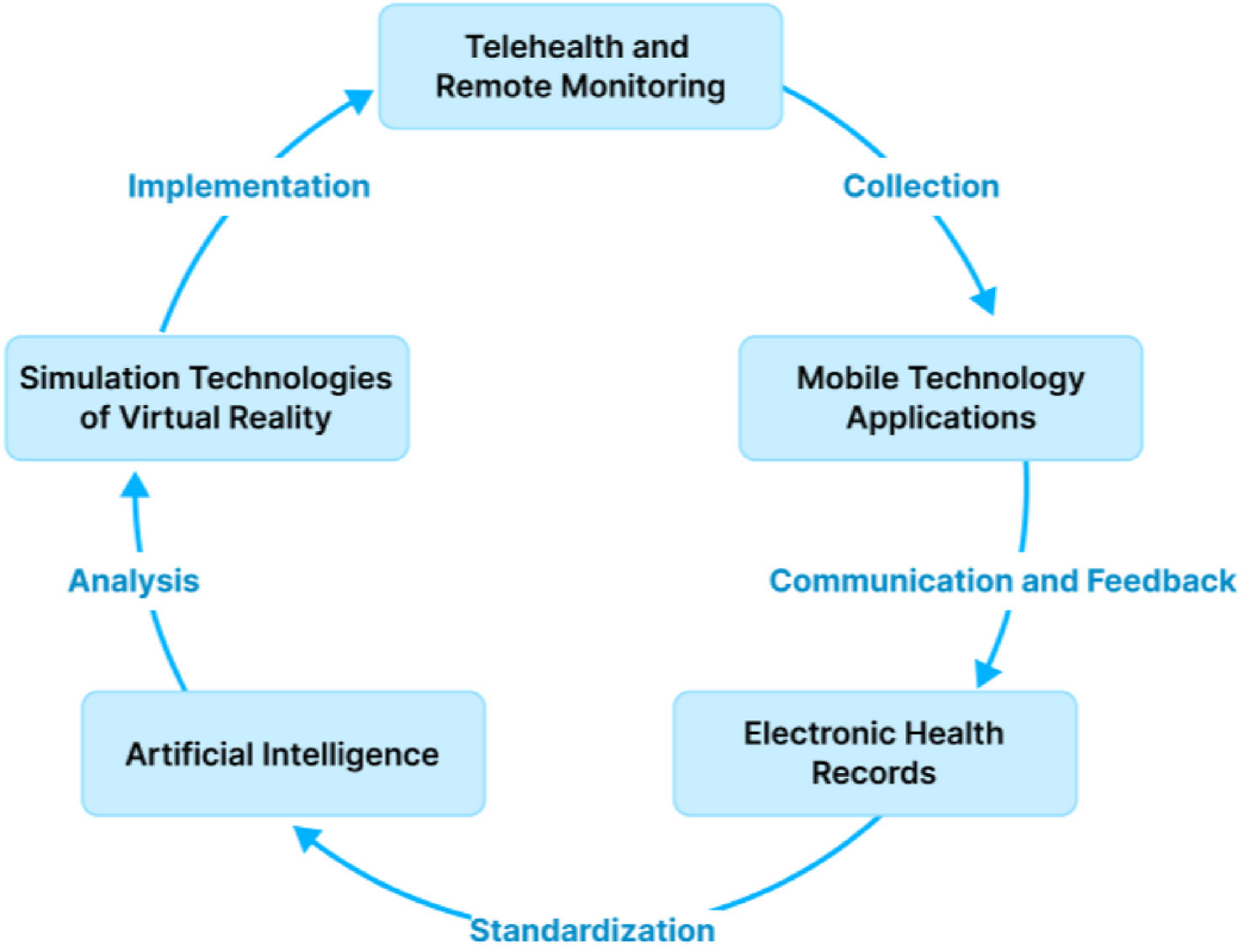
Integration of technology innovations into cancer nursing care.

## Telehealth and remote monitoring: acquisition of patient information

Due to the application of telehealth and remote monitoring technologies, traditional cancer care model is gradually shifting toward community and home-based care models. The timely acquisition of patients' health information at home is crucial for the effective promotion of home-based care for patients with cancer. On the one hand, the advent of telehealth and remote monitoring enables electronic transmission of patients' physical activity and medical data from their homes, significantly reducing the necessity for frequent hospital visits.^8^ On the other hand, this real-time data monitoring also assists nurses in early detection of patients’ health problems, and facilitates prompt decision-making, thereby enhancing the quality of cancer care.

## Mobile technology applications: bridging communication between patients and health care providers

In clinical settings, there are significant communication barriers that have existed between medical staff and home-based patients. Patients frequently encounter complex processes when attempting to contact their health care providers. Such communication barrier can lead to treatment delays and disrupt the continuity of care. Mobile technology applications act as a vital link between home-based patients and oncology nurses.^9^ By utilizing the mobile technologies, health care providers can not only provide pertinent health education information to their patients but also enable patients to obtain disease-related feedback to nurses timely. Thus, mobile technologies bridging easy communication between patients and health care providers, enhancing patients to conduct self-care management, may lead to improve their health outcomes.^10^

## Electronic health records (EHRs): enhancing the continuity of cancer care

EHRs represent digital versions of patient records, encompassing details such as patient demographics, disease specifics, and the treatment trajectory.^11^ EHRs can facilitate the identification of trends, patterns, and outcomes of cancer patient care and provide timely and reliable dynamic surveillance data, which are essential in guiding strategies in reducing cancer morbidity and mortality. Additionally, EHRs are continually updated through the exchange of health information among health care professionals during interventions. Consequently, regular usage of EHRs has been shown to minimize data fragmentation and to enhance the continuity of oncology care.^12^

## Artificial intelligence (AI) technologies: facilitating precision care

AI technologies have obtained widespread application in various aspects of cancer care including diagnosis, cancer classification and staging, assessing treatment response, and prognostication.^13^ AI can rapidly analyze diverse clinical data, offering quick solutions of data analysis. In cancer care settings, AI empowers researchers to identify trends in disease progression, accurately predict adverse complications, and develop personalized, precise interventions to improve the health outcomes of patients with cancer.^14^^,^^15^

## Simulation technology of virtual reality (VR): application scenarios in cancer care

Simulation and VR technologies offer a dynamic, interactive environment for real-time simulation. Beyond its application in medical education, VR plays a crucial therapeutic role in cancer care. Leveraging the simulated environment of VR technology, patients with cancer can engage in diverse exercise rehabilitation programs, characterized by high acceptability and feasibility, thus enhancing adherence to the exercise regimen.^16^ In addition, integrating VR technology with psychological approaches like relaxation and distraction therapies, as well as cognitive behavioral therapy, can mitigate cancer patients’ psychological stress and improve their mental well-being.

Overall, the scope of these technologies in cancer care may be broader than the description of this editorial. For instance, mobile technology applications, beyond their communicative capabilities, offer a range of functionalities including health monitoring and education. Similarly, VR technologies may be employed not only for simulation and therapy but also for assessing the cognitive functions of patients with cancer. The application of these cutting-edge technologies is indeed multidimensional. The crucial aspect lies in effectively integrating these technologies into the clinical nursing process, thereby maximizing their clinical utility in cancer care.

## Funding

This editorial was funded by the National Natural Science Foundation of China (Grant No. 72004039) for Dr Zeng.

## CRediT authorship contribution statement

**Guolong Zhang:** Writing draft. **Xuanhui Liu:** Revise manuscript. **Yingchun Zeng:** Conceptualization, Reviewing, and Editing.

## Declaration of competing interest

All authors have no conflicts of interest to declare. The corresponding author, Dr. Yingchun Zeng, is a member of the editorial board of the *Asia-Pacific Journal of Oncology Nursing*. The article underwent the journal's standard procedures and was handled independently, with no involvement from Dr. Zeng and their research groups.

## Declaration of Generative AI and AI-assisted technologies in the writing process.

No AI tools/services were used during the preparation of this work.
